# Molecular and Cellular Characterization of Human CD8 T Suppressor Cells

**DOI:** 10.3389/fimmu.2016.00549

**Published:** 2016-11-30

**Authors:** Zheng Xu, Sophey Ho, Chih-Chao Chang, Qing-Yin Zhang, Elena-Rodica Vasilescu, George Vlad, Nicole Suciu-Foca

**Affiliations:** ^1^Immunogenetics and Cellular Immunology, Department of Pathology and Cell Biology, Columbia University, New York, NY, USA

**Keywords:** CD8^+^ T suppressor cells, ILT3, co-stimulation blockade, transplantation, autoimmune disease, gene profile of CD8^+^ Ts

## Abstract

Bidirectional interactions between dendritic cells and Ag-experienced T cells initiate either a tolerogenic or immunogenic pathway. The outcome of these interactions is of crucial importance in malignancy, transplantation, and autoimmune diseases. Blockade of costimulation results in the induction of T helper cell anergy and subsequent differentiation of antigen-specific CD8^+^ T suppressor/regulatory cells (Ts). Ts, primed in the presence of inhibitory signals, exert their inhibitory function in an antigen-specific manner, a feature with tremendous clinical potential. In transplantation or autoimmunity, antigen-specific Ts can enforce tolerance to auto- or allo-antigens, while otherwise leaving the immune response to pathogens uninhibited. Alternatively, blockade of inhibitory receptors results in the generation of cytolytic CD8^+^ T cells, which is vital toward defense against tumors and viral diseases. Because CD8^+^ T cells are MHC Class I restricted, they are able to recognize HLA-bound antigenic peptides presented not only by APC but also on parenchymal cells, thus eliciting or suppressing auto- or allo-immune reactions.

## Introduction

Over the last decade, the prevailing dogma has been that self-tolerance is mediated through dominant suppression of autoimmune responses by regulatory CD4^+^CD25^+^FoxP3^+^ T cells (CD4^+^ Treg). Naturally occurring Tregs specifically express the transcription factor FOXP3 (forkhead box P3) (1). Natural CD4^+^CD25^+^ Treg constitute 5–10% of peripheral CD4^+^ T cells in normal mice and <5% in humans. Their essential role in tolerance was shown by experiments in which the depletion of natural Tregs from the thymus of newborn rodents resulted in enhanced immune responses to conventional bacteria from the intestine. This provoked inflammatory bowel disease (IBD) and the development of autoimmune diseases. In contrast, expansion of Tregs suppressed allergy, organ allograft rejection, graft-versus-host disease after bone marrow transplantation, and various autoimmune diseases (1–5).

The revival of CD8^+^ suppressor cells (CD8^+^ Ts) after decades of deliberate omission has been well described in some review articles (6–8). The function of CD8^+^ Ts was first documented in the early 80s by Gershon et al. (9). With the advent of molecular immunology, the existence of the murine I-J locus, presumed to encode Ts function, could not be confirmed. For fear of rejection and denial of grant support, the word “suppressor” was arbitrarily replaced with that of “regulatory” T cells, even though the sole function of regulators was to suppress immune function. For this reason, the reader of the suppressor literature would be well advised to search the listing of papers referring to either CD8^+^ Treg or T suppressor cells (CD8^+^ Ts).

Both CD8^+^ and CD4^+^ Tregs showed similar expression levels of FOXP3 and CTLA-4, which represent their most characteristic markers. On the other hand, the biggest difference between CD4^+^ and CD8^+^ Tregs resides in the expression of CD28 (10). CD4^+^ Tregs express a higher level of CD28, which is required for their interaction with B7 molecules. B7 molecules regulate thymic development and peripheral tolerance (11). For CD8^+^ T cells, the expression of CD28 is partially dispensable due to their reduced production of IL-2 (12–14).

## Natural and Non-Antigen-Specific CD8^+^ Treg

Similar to natural CD4^+^ Treg, CD8^+^ thymus-derived natural Tregs have also been described. Characteristically, these cells have a CD28^−^ phenotype in both mice and human (15). However, after TCR triggering, both CD4^+^ and CD8^+^ natural Treg inhibit the immune response in an antigen non-specific and MHC non-restricted manner *via* direct interaction between Treg and activated T cells. Naturally occurring CD8^+^ Treg were reported to have a CD8^+^CD25^+^CTLA-4^+^GITR^+^FoxP3^+^ phenotype and suppress in a CTLA-4- and TGF-β1-dependent manner (16).

The Qa-1-restricted CD8 alpha, alpha^+^ (TCR alpha beta^+^), population is the best characterized population of CD8^+^ natural Treg in mice. The Qa-1 molecule (homolog of HLA-E in human) presents peptides derived from the non-hypervariable domain of the TCR. These Vbeta-specific CD8^+^ Tregs interact and inhibit the activation of CD4^+^ T cells with similar Vbeta regardless of their specificity (17–20).

Study of the miRNA profile of human CD8^+^CD25^+^ natural Treg revealed 10 differentially expressed miRNAs (miR-214, -205, -509 overexpressed and miR-9, -24, -31, -155, -335, -210, and -449 under expressed), which seem to display specific regulation of FOXP3, CTLA-4, and GARP gene expression (21).

Peripheral CD8^+^ CD28^−^ Foxp3^−^ CD56^−^ non-antigen-specific Ts were reported to be easily generated and expanded by culturing CD8^+^CD28^−^ T cells in a cocktail of cytokines containing IL-2, IL-10, and GM-CSF. They were expanded without antigenic stimulation and seemed to inhibit antigen recognition, T cell proliferation, and cytotoxicity *via* IL-10 secretion (22, 23). It has been suggested that such Ts can be extracted from patients during disease remission and reinfused during disease exacerbation (24).

## Adaptive Antigen-Specific CD8^+^ Treg

Adaptive CD8^+^ Ts originate from the post-thymic T cell pool and are induced by a variety of *in vivo* and *in vitro* antigenic stimuli. Antigen-specific Treg are required for efficient suppression of T cell immune responses against MHC-bound peptides derived from auto- or allo-antigens. The best characterized Treg in this category include human CD8^+^CD28^−^, MHC class I-restricted, T suppressor, and CD4^+^CD25^+^CD45RO^+^, MHC class II-restricted, Treg cells (10). Our previous studies have demonstrated that MHC allo-restricted CD8^+^CD28^−^ Ts can be generated *in vitro* by multiple rounds of T cell stimulation in the presence of allogenic APC. Evidence has been provided that Ts develop *in vivo* from rejection-free organ allograft recipients. Antigen-specific CD8^+^CD28^−^ Ts exert their function by conditioning APC to become tolerogenic. Our studies on the mechanism of CD8^+^CD28^−^ Ts-mediated suppression revealed that they act *via* an APC bridge, inducing the upregulation of immunoglobulin-like transcript (ILT) inhibitory receptors on professional (dendritic cell and monocytes) as well as on non-professional [endothelial cells (EC)] APC (25–29).

## CD8^+^ Ts and ILT3

The induction of tolerogenic dendritic cells (DCs) was first established in 1998 by our group (26). We showed that human CD8^+^CD28^−^ Ts cells generated by multiple rounds of *in vitro* allo-stimulation interact with APC, inducing the downregulation of co-stimulatory molecules and thereby reducing their capacity to trigger CD4^+^ T helper (T_h_) cell activation (27). In the absence of T_h_ cell help, CD8^+^ T cells from the same culture acquire suppressor activity. Similarly, multiple stimulations of human T cells with xenogeneic APC or with peptide-pulsed autologous APC resulted in the generation of antigen-specific CD8^+^CD28^−^ Ts cells (28, 29). These CD8^+^ Ts cells, derived from an oligoclonal population, are MHC class I restricted and express same levels of FOXP3, GITR, CTLA-4, CD25, OX40, CD103, CD62L, 4-1BB, and TNFRII as seen in CD4^+^CD25^+^ natural T regulatory (Treg) cells (10, 30).

CD8^+^CD28^−^ Ts can be distinguished from CD8^+^CD28^−^ CTL cells from the same multiple allo-stimulated T cell line (TCL) by the higher expression of some genes from the killer cell inhibitory receptor (KIR) family, such as KIR3DL1, KIR3DL2, and KIR2DL3 and by their gene profile (10).

Upon restimulation with priming APC, CD8^+^ Ts do not produce IFN-γ, IL-10, TGF-β, or other cytokines. Instead, CD8^+^CD28^−^ Ts inhibit CD40-mediated upregulation of co-stimulatory molecules, such as CD80 and CD86 on priming APC, which become tolerogenic, upregulating the expression of the inhibitory receptors ILT3 (also called LILRB4, CD85K, or LIR5) and ILT4 (also known as LIR-2, LILRB2, or CD85d). Consequently, APC are rendered unable to induce and sustain the full program of CD4^+^ T_h_ cell activation and maturation, due at least in part to inhibition of Nuclear Factor-κB (NF-κB) activation and subsequent transcription of co-stimulatory molecules (10, 26–29, 31–34).

Tolerogenic APC can be also generated by exposure of DC to IL-10, IFN-α, or IFN-β, which induce upregulation of ILT3 and ILT4 (14–17, 31–34).

The crucial role of ILT3 and ILT4 was revealed in experiments in which the myelomonocytic cell line KG1 was transfected with ILT3 or ILT4 and used for T cell allo-stimulation. Wild KG1 cells induced strong MLC responses, while ILT3- or ILT4-transfected KG1 cells were non-stimulatory. CD8^+^CD28^−^ T cells from the same cultures inhibited autologous T cell responses to wild KG1 stimulating cells, displaying suppressor function. CD4^+^ T_h_ reactivity to KG1-ILT3 or KG1-ILT4 transfectants could be restored by adding to the cultures either anti-ILT3 or ILT4 mAb, respectively, or IL-2. These results indicated that ILT3- or ILT4-expressing APC induce T cell anergy and elicit the differentiation of CD8^+^CD28^−^ Ts (26).

Allogeneic CD40L-activated pDC (expressing high levels of ILT3 and ILT4) promote the differentiation of naïve CD8 T cells into CD8^+^ Ts. These CD8^+^ Ts inhibit T cell proliferation *via* secretion of IL-10 (35).

While overexpression of ILT3 was shown to be a marker of tolerogenicity, knock down of ILT3 augmented the immunogenic capacity of activated DC, significantly increasing their capacity to migrate, produce inflammatory cytokines, and activate IFN-γ- and IL-17-secreting T effector cells (36).

ILT3 and ILT4 belong to a family of Ig-like inhibitory receptors that are structurally and functionally related to KIRs. Some ILT family members, including ILT2, ILT3, and ILT4, have long cytoplasmic tails containing ITIM. These receptors mediate inhibition of cell activation by recruiting the tyrosine phosphatase SHP-1 (37–40). Coligation of ILT3 and ILT4 in monocytes inhibits Ca^2+^ mobilization and tyrosine phosphorylation, which is triggered by Ab ligation of FcRII (CD32), HLA-DR, and FcγRI (CD64). The ligand for ILT3 has not been described so far. ILT4 was shown to bind to the α3 domain of HLA class I (HLA-A, HLA-B, HLA-C, and HLA-G), competing with CD8 for MHC class I binding (41, 42). As a result, recombinant soluble ILT4 restores, rather than inhibits, Treg proliferation (43).

Besides the negative signaling that ILT3 transmits endogenously upon ligation, ILT3’s extracellular Ig-like domains are also endowed with inhibitory function. This was demonstrated in experiments for which we first engineered the myelomonocytic KG1 cell line (KG1-Delta) to overexpress a signaling-defective ILT3 deletion mutant which lacked the cytoplasmic tail containing ITIM. CD4^+^ T-cell responses in primary and secondary MLC were greatly deficient upon stimulation with these cells, which elicited instead the generation of CD8^+^CD28^−^ Ts cells. This result suggested that the extracellular domain of ILT3 by itself carries out a tolerogenic function which is independent of the inhibitory intracellular signaling (43). Based on these findings, we engineered a recombinant ILT3.Fc protein, which lacked both the trans-membrane and intracellular domain. When ILT3.Fc was added to T cells at the time of MLC priming, it suppressed CD4^+^ T_h_ cell proliferation and elicited the differentiation of allospecific CD8^+^ Ts *in vitro* as well as *in vivo*. Hence, both membrane and soluble ILT3 induce CD4^+^ T helper anergy, triggering the generation of CD8^+^ Ts cells (41, 44, 45). This indicates that ILT3 is an essential immune checkpoint or master switch which regulates the outcome of the immune response.

Soluble ILT3, engineered as an ILT3.Fc fusion protein, was shown to induce tolerance to allogeneic human pancreatic islet transplants in humanized NOD/SCID mice (hu-NOD/SCID) (44) and to reverse progression of rejection after its onset (34). ILT3.Fc inhibited both the cellular and humoral arm of rejection, as shown by the inhibition of T_h_1 and T_h_2 proliferation and cytokine production, CTL generation, and synthesis of anti-HLA and xenospecific antibodies by B cells from tolerant animals (34, 44, 46).

## Gene Profile of ILT3.Fc-Induced CD8^+^ Ts

ILT3.Fc dramatically changes the landscape of the gene expression profile in CD8^+^ Ts cells. Numerous genes in the WNT receptor pathway were significantly upregulated, indicating its important role in the generation of CD8^+^ Ts cells. This data support the concept that activation of the WNT pathway inhibits CD8 T-cell proliferation and cytotoxic effector cell differentiation. The expression of TGF-β and TGFBR2 was also significantly increased, consistent with the well-characterized cross talk between TGF-β and WNT pathway (47, 48).

ILT3.Fc extensively downregulated the expression of cyclins and cyclin kinases while upregulating cyclin-dependent kinase inhibitors. Considering the fact that cyclins and cyclin kinase together with their specific inhibitors are the most important positive and negative regulators in the cell cycle, it is reasonable to assume that ILT3.Fc induces cell cycle arrest, inhibiting T cell proliferation (47, 49).

On the gene transcriptional level, ILT3.Fc promotes the expression of transcriptional repressors which block the synthesis of cytokines and other factors necessary for T cell proliferation and differentiation. The zinc finger transcriptional repressor BCL6 is one of the genes whose elevated expression is important for the differentiation of ILT3.Fc-induced Ts. We found that transfection of BCL6 in allo-activated CD8^+^ T cells converted them into suppressors, whereas silencing of BCL6 in unprimed T cells prevented their differentiation into Ts when allo-stimulated in the presence of ILT3.Fc. BCL6-transfected Ts share highly similar characteristics with ILT3.Fc-induced Ts both *in vitro* and *in vivo*. The *in vivo* evidence is based on the finding that BCL6 was overexpressed in human CD8^+^ T cells from humanized mice rendered tolerant to pancreatic islet transplants by treatment with ILT3.Fc (34). ILT3.Fc-induced repression of granzyme B, IFN-γ, IL-5, and enhancement of CXCR4 occurred in conjunction with the upregulation of BCL6 expression in CD8^+^ Ts cells. Hence, ILT3.Fc may arbitrate T cell lineage fate through BCL6-mediated repression of T_h_1, T_h_2, T_h_17, and CTL and induction of Ts differentiation (34).

MiRNA represents a group of novel regulatory molecules which modulate gene function at the posttranscriptional level. Studies on the miRNA expression profile in ILT3.Fc-induced CD8^+^ Ts indicate that they also play a role in the generation of CD8^+^ Ts cells. ILT3.Fc inhibited the expression of miR-21, miR-30b, and miR-155. Those miRNAs target the 3′-untranslated region of DUSP10, BCL6, and SOCS1, genes whose transcription was highly increased in ILT3.Fc-induced Ts.

Primed CD8^+^ T cells transfected with miR-21 and 30b, miR-21 and 155, or miR-21, 30b, and 155 inhibitors displayed suppressor activity when added to autologous CD3-triggered CD4^+^ T cells. Luciferase reporter assays of miR-21 and miR-155 indicated that their transcription is highly AP1 dependent, consistent with the finding that for the AP1 subunits, FOSB, and c-FOS, translocation to the nucleus is inhibited by ILT3.Fc. In summary, ILT3.Fc inhibits T cell activation and induces the generation of Ts by targeting multiple inflammatory miRNA pathways (50). Recent studies on human natural CD8^+^CD25^+^FOXP3^+^CTLA-4^+^ Treg cells revealed similar miRNA signatures. The data indicate that miRNAs, including miR-9, -24, -155, and -335, play an important role in the induction of CD8^+^ Treg by modulating Treg-associated genes (21).

Studies of exosomes from MLC supernatants revealed the presence of inflammatory microRNA, including miR-146a, miR-155, miR-21, miR-30b, miR-365, and Let-7a. These miRNAs were inhibited when ILT3.Fc was added to the culture, they were produced exclusively by CD4^+^ T cells, being absent from CD4-depleated cultures. Furthermore, upon treatment with exosomes containing inflammatory microRNA, ILT3.Fc-induced CD8^+^ Ts lost their suppressive activity at low Ts/T effector cell ratio (51).

MiRNAs contained by exosomes released from allo-activated T cells enhanced T helper activity even in the presence of limiting amounts of allospecific T suppressor cells. This suggests that increased amounts of microRNA in recipients’ sera may serve as markers of active immune responses against the graft, even in the presence of regulatory T cells. Furthermore, such exosomes may be of use in eradicating tumors in patients developing lymphoid malignancies secondary to immunosuppression and viral (EBV, CMV, Hepatitis B and C) infections (51).

Comparison of ILT3.Fc-induced CD8^+^ Ts with CD8^+^CD28^−^ Ts induced in MLC by chronic allogenic stimulation demonstrated that the characteristic signatures of CD8^+^ T suppressor cells generated by either of these methods are the same, consisting of upregulation of the BCL6 transcriptional repressor and downregulation of inflammatory microRNAs, miR-21, miR-30b, miR-146a, and miR-155. In conclusion, microRNAs, which are increased under inflammatory conditions in activated CD4^+^ and CD8^+^ T cells with helper or cytotoxic function, show low levels of expression in CD8^+^ T cells that have acquired antigen-specific suppressor activity (52).

## CD8^+^ Ts in Transplantation

The possible role of ILT3 and ILT4 molecules in maintenance of quiescence in transplant patients is of obvious interest. We found that T cells from heart and liver transplant patients in quiescence, but not from recipients with a history of rejection episodes, induced the upregulation of ILT3 and ILT4 and downregulation of CD80 and CD86 in cryopreserved APC from the donor. As a surrogate for donor APC, DC matched to the donor for at least one HLA class I and one HLA Class II (DR) can be used for flow cytometry and functional assays.

Monitoring of kidney allograft recipients who have been chronically exposed to rapamycin showed increased numbers of DC with the ILT3^+^ILT4^+^ tolerogenic phenotype and of T cells with the CD8^+^CD28^−^ suppressor phenotype suggesting that mTOR inhibition promotes a novel immunoregulatory pathway (53).

However, since donor DC migrate out of the graft early following transplantation, it was still unclear how quiescence was maintained by some, but not all organ allograft recipients. The most likely explanation seems to be that graft EC, which are non-professional APC that express all donor HLA allo-antigens, become tolerogenic. To explore the possibility that EC are targeted by recipient CD8^+^CD28^−^ Ts, we transfected umbilical cord EC (matched to the donor for at least one HLA class I antigen) with luciferase ILT3 or ILT4 reporter gene and performed luciferase transcription assay in the presence of recipient CD8^+^CD28^−^ T cells. These experiments demonstrated that CD8^+^CD28^−^FoxP3^+^ T cells from the circulation of rejection-free heart transplant patients triggered the expression of ILT3 and ILT4 in EC-sharing class I HLA antigens with the graft. CD8^+^ T cells from patients with recurrent episodes of acute or with chronic heart allograft rejection did not display ILT3-inducing capacity (54, 55). Using cell fractionation and sequencing studies, we further showed that ILT3 precursor RNA are expressed and retained in the nuclei of resting EC. Ts interaction with EC or exposure of EC to IL-10 and IFN-α triggers processing of ILT3 pre-mRNA. Western blot analysis showed that the expression of the mature ILT3 transcript is accompanied by production of ILT3 protein (56). Studies from other laboratories further confirmed our finding that IL-10 also inhibits endothelium-dependent T cell co-stimulation by upregulating ILT3 and ILT4 in human vascular EC (57).

The tolerogenic role of EC, which express inhibitory molecules, was further explored in a Lewis to ACI rat heart transplantation model. After three injections of UV-irradiated blood from Lewis donors, about 50% of the ACI recipients became tolerant to donor strain heart transplants. Tolerance could be transferred to secondary ACI recipients by CD8^+^ but not by CD4^+^ T cells. Furthermore, the graft of these secondary recipients was tolerated indefinitely even when transplanted to tertiary, non-conditioned ACI recipients. The CD8^+^ T cells used for adoptive transfer of tolerance were FoxP3^+^. They induced the expression of PIR-B, a rat ortholog of ILT4, not only in donor APC, but also in the EC lining the aorta of the transplanted heart. Hence, this phenomenon of “graft adaptation” was mediated by the induction of inhibitory receptors in graft EC by MHC Class I allo-restricted CD8^+^ suppressor cells (58).

Recently, it was shown that kidney–pancreas transplantation in a type I diabetic patient was characterized by an increased presence of CD8^+^CD28^−^ Treg in the pancreas and elevated levels of ILT3 expression on APC in a donor-specific manner (59).

Our conclusion that generation of allospecific CD8^+^CD28^−^ Ts may require the induction of anergy in CD4^+^ T helper cells (T_h_), e.g., leaving the primed CD8^+^ T cells “helpless,” is supported by other groups. These investigators used the same MLC stimulation model, but “allo-anergized” T_h_ by adding to the MLC a CTLA-4 immunoglobulin fusion molecule (Belatacept) which blocks the CD28-B7 co-stimulation pathway. They further confirmed our finding that repeated rounds of allo-stimulation results in relative and absolute expansion of CD8^+^Foxp3^+^ Ts. Finally, they found that allo-anergized CD8^+^CD28^−^ Ts of donor origin are expanded in recipients of allogeneic hematopoietic stem cell transplantation (60). This finding is reminiscent of our and other authors’ previous observation that CD8^+^CD28^−^ Ts are present in the circulation of transplant recipients in quiescence (26, 61–63).

Human allo-antigen-specific CD8^+^ Tregs were generated in a large scale from antigenically naïve precursors by *in vitro* stimulation with allogeneic CD40 activated B cells. These cells inhibited GVHD in a humanized mice model, suppressing allo-reactive T cell proliferation and cytokine production by a CTLA-4-dependent mechanism (64). In other studies, CD8^+^CD28^−^Tregs were generated in MLC by coculture with mesenchymal stem cells, a method of potential use given the resistance of CD28^−^ T cells to treatment with Belatacept, an agent which blocks CD28/B7 co-stimulation, preventing alloreactivity in kidney transplant recipients (65).

In recent studies, CD8^+^CD28^−^ Ts cells were generated by multiple MLC simulation and expanded by adding common gamma chain cytokines IL-2, IL-7, and IL-15 to the cultures. The expanded population exhibited increased expression of CTLA-4, FOXP3, and CD25, while the expression of CD56, CD57, CD127, and perforin was downregulated (66). Consistent with our own studies, suppression of CD4 T_h_ by CD8^+^CD28^−^ Ts was HLA class I allo-restricted and cytokine independent. However, the claim that after expansion these cells keep their suppressor function without killing the stimulating APC, as demonstrated in CFSE cytotoxic assays, could not be confirmed in our own studies for which the traditional Cr-51 release from target cells was used. We found that when these cytokines were used for expansion, the proliferating population consisted of CD8^+^ CTL. It is apparent that rather than being in a terminal stage of differentiation, CD8^+^CD28^−^ Tcells can be expanded indefinitely given the appropriate mixture of cytokines (67). A similar phenomenon has been described for CD4^+^. Zhou et al. have shown that CD4^+^ Tregs constitute an unstable T cell subset that can be reprogramed into pathogenic effector cytokine-producing T cells. Such CD4^+^ T cells downregulate their FOXP3 expression, losing suppressive capacity and acquiring an activated-memory phenotype (68). These findings reflect the plasticity of both CD8^+^ and CD4^+^ Treg cells, which can revert their function from suppressors to effector cells. A comprehensive review of CD8^+^ Treg in animal transplantation studies can be found in Guillonneau et al., which also emphasizes the identification of PD-1 as a marker of CD8^+^ Ts in rodents (69).

Recently, a new subset of CD8^+^CD122^+^PD1^+^ Tregs, which produce IL-10, IFN-γ, and TGF-β and suppress CD4^+^ T cell activation, has been described in mice. Its counterpart in human is still unknown (70). This CD8^+^cd122^+^ Treg subset was claimed to more potently suppress allograft rejection compared to their CD4^+^CD25^+^ Tregs (71). Therefore, it appears that CD8^+^CD28^−^, CD8^+^Qa-1^+^, CD8^+^CD103^+^, and CD8^+^CD122^+^PD-1^+^ Treg subsets may share the capacity of maintaining homeostasis with an equally heterogeneous population of CD4^+^ Tregs.

## CD8^+^ Ts Cells in Autoimmune Diseases

CD8^+^ T cells can oppose or promote autoimmune disease through activities as suppressor or as cytotoxic effectors (72). Data have been presented that CD8^+^CD28^−^CD56^−^ T cells have suppressive activity in rheumatoid arthritis (RA), preventing the activation of naïve CD4^+^ T cells and inhibiting their effector function *in vivo*. When transferred into NOD-SCID chimeras engrafted with human synovial tissue, they suppressed the inflammatory activity in the synovial lesions and inhibited cytokine production (73). Rheumatoid synovitis could also be treated in such chimeras by infusing autologous CD8^+^CD16^+^ T cells, which inhibits the production of IL-1β, IFN-γ, TNF-α, and other inflammatory cytokines. Treatment with IL-16 mimicked the effect of the adoptive transfer of Ts and anti-CD16 antibodies abrogated the suppressor effect (74). Similarly, there is evidence that CD8^+^ Ts generated from SLE patients during remission had suppressor activity, while CD8^+^ obtained during exacerbation of the disease had no such activity (75).

Glatiramer acetate (GA) introduced in the therapy of multiple sclerosis has been shown to induce CD8^+^ Ts, which seem to recognize GA on the cell surface and directly kill CD4^+^ T cells in a HLA-E-dependent manner (76). Ulcerative colitis and Crohn’s disease are other examples of pathological processes in which CD8^+^ Ts may inhibit proliferation of CD4^+^ T cell through a TGF-β-dependent mechanism (77). Intestinal epithelial cells may activate CD8^+^ Ts cells, which downregulate IBD, suppressing IgG production (78). In JDM, a one-course administration of humanized anti-CD3 mAb was claimed to induce the generation of CD8^+^ CD25^+^CTLA-4^+^FoxP3^+^ Ts cell, which were able to inhibit the stimulation of CD4^+^ T cells in *in vitro* coculture system (79, 80). Accumulating evidence indicates that co-inhibitory molecules, such as CTLA-4, PD1, and BTLA, are negative regulators of immune responses since deficiencies or mutations result in the development of autoimmune diseases. The administration of decoy co-inhibitory receptors, such as CTLA-4.Ig or agonistic antibodies, can suppress the response of self-reactive T cells in autoimmune diseases.

Abatacept, a fusion protein composed of the Fc fragment of human IgG1 linked to the extracellular domain of CTLA-4, has shown efficacy in a broad spectrum of RA patients from early stage to refractory diseases that are resistant to TNF blockers (81–84).

In addition, Abatacept showed efficacy in patients with juvenile idiopathic arthritis, psoriasis, and SLE. CTLA-4 competes with CD28 on the membrane of activated T cells for binding to B7 molecules (CD80 and CD86) on APC, delivering negative signals which inhibit or terminate T cell responses.

PD1, another co-inhibitory receptor from the CD28 family, which is expressed on activated T cells (as well as on B cells and monocytes), binds to two ligands of the B7 family, PD-L1 and PD-L2. The ligation of PD-1 with these ligands inhibits CD4^+^ and CD8^+^ T cell proliferation by arresting the cell cycle (85–87). However, in human, PD1 ligation is important to inhibition of cell proliferation and death by apoptosis, rather than to the generation and function of CD8^+^ Ts.

BTLA-HVEM pathway is another inhibitory pathway for lymphocyte activation [reviewed in Ref. (78)]. BTLA4, a member of the TNF family, is expressed on CD4^+^ and CD8^+^ T cells, NK and NKT cells, as well as on B cells, DC, and macrophages. Its ligand HVEM is also widely expressed on hematopoietic cells, including T cells and APC. Ligation of BTLA induces its tyrosine phosphorylation and SHP-1/SHP-2 association, inhibiting T cell proliferation and IL-2 production. Deficiency of BTLA-HVEM interaction has been shown to be involved in autoimmune diseases, though no clinical trial is yet in progress.

Although current methods of immunotherapy in cancer are largely based on the use of the immune checkpoint inhibitors anti-CTLA-4 and anti-PD-1 antibodies, the major obstacle resides in opportunistic autoimmune disorders and associated morbidity resulting from altered immune regulation (88, 89).

Other lymphocyte subsets believed to have suppressor function in autoimmune diseases and transplantation include CD3^+^CD4^−^CD8^−^ (double negative Treg), CD4^+^, Valpha14 negative (NKTreg), and gamma/delta Treg cells. It was postulated that there are four modes of Treg function: (1) secretion of inhibitory cytokines such as IL-10 and TGF-β; (2) granzyme-perforin-induced apoptosis of effector T lymphocytes; (3) induction of apoptosis by deprivation of cytokines; and (4) inhibition of DC function (8, 90).

Specific recognition of the MHC Class Ib Qa-1bound peptides expressed on activated CD4 T cells by regulatory, cytolytic CD8^+^ T cells was postulated to prevent autoimmunity in mice (91). A similar function has been attributed to human neuroantigen-specific CD8^+^ Treg, which recognize HLA-E-bound peptides and are present in the circulation of patients with multiple sclerosis (92).

A distinct subset of human CD8^+^CD25^+^FoxP3^+^ Treg seems to be characterized by the expression of the lymphocyte activation gene-3 (LAG-3). The suppressive activity of this subset has been attributed to the secretion of the CC chemokine ligand 4 (CCL4), which interferes with TCR signaling, inhibiting T cell activation. These Tregs can be expanded only from T cells primed *in vivo* to a specific antigen by repeated or chronic stimulation *in vitro* or *in vivo*, respectively. This indicates that they are adaptive Treg (90).

It has been shown that autologous hematopoietic stem cell transplantation in refractory SLE can induce immunological tolerance to auto-epitopes from nucleosomes by restoring the CD8^+^FoxP3^+^ TGF-β producing pool of suppressors. These Ts maintained high expression levels of latency-associated peptide (LAP), CD103, PD-L1, and CTLA-4 following transplantation and completely inhibited autoimmunity (93).

Collectively these data indicate the importance of CD8^+^ Treg in suppressing autoimmune responses and transplant rejection.

## CD8^+^ Regulatory T Cells in Persistent Viral Infection

The control of virus-specific immune responses may be a mechanism that permits virus persistence. On the other hand, it may also protect the patient from overwhelming T cell reactivity and destruction of infected tissues (94).

In patients with HIV, it was shown that stimulation of patients’ PBMC with HIV-specific antigens induced TGF-β-producing CD8^+^ Treg. These CD8^+^ Treg suppressed the IFN-γ production of HIV-specific and vaccinia virus-specific CD8^+^ T cells, displaying both a specific and non-specific activity. IL-10-producing CD8^+^ Treg were also expanded from the peripheral blood of HIV-infected individuals and their frequency seems to be associated with impairment of CD8^+^ effector-cytolytic function (95–97).

Distinct populations of CD8^+^ Ts were also shown to be present in the blood and liver of patients with chronic HCV infection. Some investigators reported the expansion of CD25^−^FoxP3^−^CD8^+^ Treg that inhibited IFN-γ production of HCV-specific CD4^+^ and CD8^+^ T cells *via* TGF-β production. A population of HCV-specific FoxP3^+^CD8^+^ Treg was also expanded by stimulation with HCV peptides of PBMC from patients with chronic HCV infection. These cells inhibited in an antigen non-specific manner, T cell proliferation *via* direct T cell–T cell interaction (98, 99). Similarly, expansion of virus-specific CD8^+^BTLA^+^ T cells in the liver was observed in patients with chronic HBV infection. These infiltrating regulatory T cells were antigen specific and suppressed T cell responses *via* IL10 secretion (100).

Intra-hepatic IL-10-secreting CD8^+^ Treg were described to be present in patients with chronic infection and to suppress IFN-production of effector CD8^+^ T cells primed to the same HCV peptide. Blockade of IL-10 restored effector activity (101).

Herpes viruses, such as EBV, CMV, or HSV, which infect many people worldwide, establish persistent latent infections which, upon reactivation in immunological deficient individuals, are responsible for life threatening episodes of infection. EBV-specific CD8^+^FoxP3^+^ Treg cells produce IFN-γ and IL-10 but not TGF-β and suppress CD4^+^ T cell proliferation in a cell–cell contact manner. Similar mechanisms seem to occur in CMV infection (102, 103).

Evidence has been provided that dermal CD14^+^ DCs, which express ILT2 and ILT4, prime a fraction of naïve CD8^+^ T cells that produce type 2 cytokines (IL-4 and IL-5) as opposed to Langerhans cells, which have no ILTs and are highly efficient in priming CD8^+^ CTL. The ILT molecules on dermal DC polarized the T cell response toward type2 cytokine producers, as blocking of these receptors enhanced the generation of CTL (104).

Accumulating evidence revealed that viruses have evolved strategies to evade the immune surveillance by inducing specific Tregs. Novel strategy show promising antiviral effects by deletion or inactivation of viral-induced Tregs cells (105).

## Conclusion

In light of the multiple pathways which may lead to the activation, generation, and expansion of T cells with antigen-specific function, it is obvious that therapy based on enhancement or blockade of immune checkpoints used by T cells for interacting with other cells holds promising results. Recombinant ILT3.Fc, CTLA-4.Ig, PD-L1.Ig, or humanized monoclonal antibodies which block effector–ligand interaction are only examples of the numerous agents that may have beneficial effects. However, the success of novel therapies aimed at suppression of autoimmune diseases depends on progress in certain areas:

1. It is apparent that inhibition of immune responses to unidentified autoantigens, deriving from a variety of tissues, calls for a better understanding of signaling pathways activated by ligation of different co-inhibitory checkpoints. This may allow for the design of combination therapy in which agents which act in synergy can be used to inhibit the activation and maturation of effector T helper and cytotoxic cells.
2. Studies on mechanisms’ underlying memory of both effectors and suppressors of T cell immune responses to defined antigens may permit the design of clinical protocols which maintain quiescence in patients in remission.
3. Identification of markers that characterize different stages of T cell conversion from one effector function to another, as exemplified by CD8^+^ suppressor and cytotoxic T cells, as well as of the mechanism of such a transition, is required for patients’ monitoring and better timing of therapy.
4. Progress in understanding the way in which cells communicate with each other to perceive endogenous and exogenous signals may open new horizons to immunotherapy of autoimmune diseases and of cancer.

## Author Contributions

ZX drafted the paper, organized its content, and collected and summarized the data. SH participated in the editorial process, data collection, and analysis. C-CC, Q-YZ, E-RV, and GV performed the critical revision of this article. NS-F is the PI of the study and made the ultimate decision on the manuscript.

## Conflict of Interest Statement

The authors declare that the research was conducted in the absence of any commercial or financial relationships that could be construed as a potential conflict of interest.
